# mHealth Applications for Childhood Anxiety Disorders: Current Landscape, Challenges, and Future Directions

**DOI:** 10.1007/s11920-025-01613-9

**Published:** 2025-05-09

**Authors:** Cigdem Sari Ozturk, Kadriye Demir

**Affiliations:** 1 Nursing Faculty, Department of Pediatric Nursing, Gazi University, Ankara, Türkiye; 2 Health Science Faculty, Department of Nursing, Lokman Hekim University, Ankara, Türkiye

**Keywords:** Childhood anxiety disorders, mHealth applications, Digital mental health tools, Pediatric mental health, App evaluation models, Privacy in digital health

## Abstract

**Purpose of Review:**

We review the literature on mHealth applications for childhood anxiety disorders, highlighting current use, limitations, and future directions.

**Recent Findings:**

Although mHealth apps targeting childhood anxiety disorders have recently increased, most have not been evaluated for clinical effectiveness, data security, or quality standards. Many apps do not contain scientifically based content and do not adequately incorporate the principles of exposure-based cognitive behavioral therapy. In addition, many apps have significant deficiencies in age-appropriateness, data security, privacy, cultural sensitivity, and accessibility. Limitations remain in terms of the standards for evaluating apps in pediatric populations.

**Summary:**

While interest in mHealth applications for childhood anxiety disorders is increasing, more research is needed to develop effective, safe, and age-appropriate digital interventions. Priorities include scientific grounding, privacy, equitable access, gamification, and parental involvement. Studies in this direction will increase the quality of applications and strengthen equality in access to mental health services.

## Introduction

Anxiety disorders are among the most common childhood mental health problems. According to the Centers for Disease Control and Prevention (CDC) 2025 report, anxiety disorders continue to be the most commonly diagnosed mental health condition in U.S. children ages 3 to 17 [1]. Alarmingly, their prevalence has been steadily increasing, particularly in adolescents and young adults, and this trend appears to have accelerated with the effects of the COVID-19 pandemic [2, 3]. Generalized anxiety disorder, social anxiety disorder, and separation anxiety disorder are common in the pediatric population. Anxiety disorders, particularly in adolescents, are associated with adverse outcomes such as poor quality of life, social and academic problems, and increased financial burden [4, 5].

Cognitive behavioral therapy (CBT), one of the evidence-based approaches to treating anxiety, is widely used, and young adults, in particular, prefer psychotherapy to psychiatric medication [6]. However, families and adolescents may experience problems in terms of access to treatment, continuity, and effectiveness for young children [7]. These difficulties include lengthy treatment protocols, lack of time and resources, limited numbers of CBT-trained therapists, and high costs. In addition, children find it challenging to complete therapy tasks at home, which can have a negative impact on treatment effectiveness [8, 9].

Recent advances in technology-based applications offer significant opportunities to overcome barriers in the treatment of pediatric anxiety disorders. These applications are reviewed under the headings of mHealth and eHealth. eHealth’ includes interventions such as the Internet, digital games, virtual reality, and robotics. At the same time, ‘mHealth’ refers to mobile and wireless applications, including text messaging, apps, wearable devices, and social media platforms [10]. With the proliferation of smartphones, tablets, and other personal electronic devices, interest in mHealth has grown exponentially over the past two decades [11].

Some of the advantages of mHealth applications in the treatment of children and adolescents include the elimination of time and space constraints [12], cost-effectiveness [7], and the potential to reduce the risk of stigmatization [13]. In addition, mHealth applications in pediatric anxiety disorders are noteworthy because they allow for individual customization and are attractive to a young population receptive to technological developments [14].

Recent studies have shown the effectiveness of mHealth apps in pediatric anxiety disorders [14–17]. However, evidence specific to children and adolescents is still limited, as most existing studies have been conducted in young adult populations. Nonetheless, some studies suggest differences in the effectiveness of interventions among adolescents aged 18–25 years and children under 18 years [18, 19]. Most existing randomized controlled trials include heterogeneous samples regarding symptom severity, diagnostic status, and age, often without stratified or subgroup analyses. Determining how interventions work for specific subgroups is difficult based on age, diagnosis, or symptom level [20, 21]. Another important issue is that adverse effects are either under-reported or not explicitly addressed in most published reports of interventions [19].

The limited scientific evidence on mHealth applications developed for pediatric anxiety disorders and the increasing number of commercial applications on the market make the knowledge gaps in this area even more important. An international study found that 94.8% of 1299 commercial mHealth applications reviewed were not based on scientific evidence [22]. It also found that these applications pose serious privacy and security risks (e.g., lack of clear information about data encryption, third-party data sharing, and the purpose of data collection) [14]. The lack of standards and regulations for mobile health applications is a global concern, especially regarding the personal safety of young people and the spread of misinformation [23, 24].

This study aims to explore the role and importance of mHealth applications developed for common childhood anxiety disorders. The first aim is to identify and describe the range of existing mHealth applications specifically designed for children and adolescents with anxiety. The second aim is to examine the characteristics of these applications, including their therapeutic foundations, design elements, and user engagement strategies. The third aim is to review the standards and tools used to evaluate their effectiveness and quality. Additionally, the study aims to address issues related to access and user interaction, as well as critical concerns surrounding data security and privacy in pediatric populations.

## mHealth Applications for Paediatric Anxiety Disorders

Mobile health (mHealth) applications are tools that provide digital support for pediatric anxiety disorders and are becoming increasingly popular [25]. These applications include emotional support tools designed to help users reframe negative thought patterns, mood trackers regularly monitoring emotional states [26], mindfulness apps offering guided exercises for staying present [14], and other features promoting behavior change. Inspired by psychotherapy models such as CBT and Mindfulness-Based Cognitive Therapy (MBCT), this content can be integrated into therapy or used independently [27]. These applications are accessible and engaging, particularly through child-friendly visuals, gamification elements, and age-appropriate language [28, 29]. The applications were examined in terms of their therapeutic foundations, design elements, and user engagement strategies to better understand their overall characteristics.

Current mobile health (mHealth) applications addressing anxiety can be classified into three main categories, although few are specifically designed for childhood anxiety disorders. The largest group includes hundreds of general applications easily accessible through the App Store, Google Play, and various internet searches. However, these applications often do not specify an age range or are aimed directly at adults [30]. Most mobile health applications focus on general anxiety or stress levels rather than a specific anxiety disorder and offer guided breathing exercises and relaxation techniques. However, this content is not fully compatible with exposure-based CBT, which is particularly effective in children [31]. Therefore, most current applications do not meet the criteria for evidence-based treatment for children [32]. Age-appropriate content, gamification, parental involvement, and structured exposure techniques should be considered together for effective mHealth solutions [33].

The second group consists of limited mHealth applications developed based on CBT principles. One of the most prominent examples in this group is SmartCAT 2.0. The application is on the Android platform and is designed to integrate with face-to-face therapy, providing structured content [34, 35]. The application includes a web-based system that encourages children to use the skills they learn in therapy daily while allowing therapists to monitor and reward this process [36]. It also provides secure two-way communication with children through the integrated clinician portal. A study conducted with 9-14-year-olds reported that users used the application an average of 12 times between sessions and reported high satisfaction levels [34].

*MindClimb* is generally compatible with CBT principles and includes exposure activities, pre-and post-assessments, and a reward system [37]. Positive feedback and points are given to the user for each completed exposure ladder in the application. However, the exposure-focused content in these applications may be overshadowed and secondary to techniques such as relaxation or cognitive restructuring. This may lead children to turn to techniques that provide short-term relaxation rather than exposure-based CBT, which has been shown to have long-term effects [38]. Therefore, exposure-based components should be presented more clearly, age-appropriately, and systematically in mHealth applications for children [36].

The third group includes mHealth applications that focus directly on exposure-based interventions. These applications usually target a specific anxiety disorder [30]. For example, *Live OCD Free* provides exposure-based content for the management of obsessive-compulsive disorder (OCD) symptoms for both children and adults. *Anxiety Coach* is a tool that supports exposure-based CBT applications for pediatric anxiety disorders, working from a database of standard exercises and allowing therapists to add personalized tasks [39]. Another example is *Lumi Nova*, a mobile application for children aged 7–12, incorporating exposure-based CBT techniques [28]. It allows children to set personal goals, follow a step-by-step exposure process, and assess their anxiety levels. In a preliminary study involving 120 participants, significant reductions in anxiety symptoms were reported after eight weeks of use. Although the study did not include a control group, the intervention yielded a medium effect size (Cohen’s d = 0.59) [28]. With its gamification elements and user-friendly design, the application stands out as an effective and reusable early intervention tool.

After reviewing examples in the literature, we see that applications focus on a specific anxiety disorder and evidence-based practices are not sufficiently integrated into the content development process [13, 28]. In this context, exposure-based mHealth applications that are individualized and socially inclusive (i.e., designed to accommodate children from diverse cultural, linguistic, socioeconomic, and ability backgrounds) are still limited. Existing applications generally encourage children to self-assess, report symptom levels, or share feelings. However, randomized controlled trials that strongly support the effect of exposure-based components are not yet available.

Another aspect that remains underdeveloped in many mHealth applications is structured parental involvement, which is particularly important for younger children. Parental participation supports app usage, goal setting, monitoring, and completing exercises outside the digital environment [40]. Recent evidence suggests that such involvement improves treatment adherence, symptom monitoring, and perceived support [40–42]. For instance, the *Rational Parenting Coach (RPC)* mobile app supports both parent and child mental health through evidence-based content, including psychoeducational materials, positive parenting strategies, feedback tools, interactive exercises, gamified sections, and guided home practice [41]. Similarly, the *Supportive Parenting for Anxious Childhood Emotions (SPACE)* program aims to reduce family accommodation and improve outcomes for both children and parents [43]. Given this evidence, integrating developmentally appropriate and structured parental components should be a key priority in designing and evaluating mHealth applications for pediatric anxiety disorders.

### Efficacy and Effectiveness of mHealth Apps: Evidence, Limitations, and Recommendations for Improvement

Although research on the use of mobile health apps for childhood anxiety disorders is increasing, the extent to which these interventions are practical and functional is not yet clear [44]. In particular, appropriately presenting exposure-based content to children is critical for both short-term effects and long-term sustainability [45].

In this sense, the extent to which applications are preferred and limited in clinical practice is an important area of debate. Uncertainties remain regarding mHealth applications in childhood anxiety disorders in terms of clinical and cost-effectiveness, preference over traditional therapies, and acceptance as a mental health service [45]. Although the available evidence is limited, some studies suggest that mobile interventions may help reduce anxiety symptoms and improve coping mechanisms in children and adolescents. Recent reviews and meta-analyses of the effectiveness and efficiency of mHealth applications developed for mental health reported an increase in the number of children not meeting diagnostic criteria and a decrease in symptoms after using the application in children diagnosed with anxiety [13, 45]. However, despite these positive findings, the quality of the research and methodological limitations are noteworthy. It has been noted that the studies included in evidence-based reviews are methodologically weak, have small sample sizes, and test the effectiveness of apps in combination with other interventions [15, 46].

Most existing studies found that mHealth applications were not sufficiently sensitive to cultural and individual differences and did not include cost-effectiveness analyses [12, 39]. Applications typically include low-intensity CBT components and limited exposure-based interventions [15, 46]. In addition, clinical support and therapist interaction are limited, and reinforcement mechanisms such as rewards or feedback that can increase user participation are often lacking [13, 46]. For all these reasons, there is insufficient evidence in the literature on the effectiveness and efficiency of mHealth applications in the care of individuals diagnosed with childhood anxiety disorders [13, 45].

Although traditional CBT is effective for childhood anxiety disorders, it is not sustainable for many families for several reasons (difficulty of access, long sessions, etc.) [34, 45]. Therefore, mobile support applications are increasingly recommended in addition to short-term face-to-face therapies. Hybrid models offer positive outcomes such as improved diagnosis, symptom reduction, and development of CBT skills, as well as clinical and cost benefits [34].

Recommendations for increasing the effectiveness of mHealth applications in childhood anxiety disorders in the literature are listed below [13, 15, 44, 46, 47].

- Application developers, researchers, and health professionals should work together to develop mHealth solutions that are evidence-based, culturally sensitive, and accessible, and these applications should be tested in methodologically sound studies [13, 47].
- To increase the effectiveness of mHealth interventions, children’s feedback should be sought during the design, testing, and post-implementation evaluation phases [15, 44].
- mHealth applications developed specifically for children should include sensor-based personalization features, a balance between simplicity and information, and more gamification [13, 46].
- mHealth applications tailored to specific anxiety disorders should be developed, and social support and clinical integration features should be added to these applications [13, 44].

The limited evidence calls for careful assessment of the content of the applications and the standards of quality and reliability.

## The Need for Quality Assessment of mHealth Apps

There are doubts about mental health apps’ scientific reliability, marketing language, and content quality. The information presented to consumers may be misleading [48]. Most existing mHealth apps for anxiety are not consistent with scientific therapeutic principles, and very few apps are compatible with exposure-based CBT. In addition, most apps are adult-focused, only include relaxation, hypnosis, and breathing exercises, and do not target a specific anxiety disorder [30]. Strong scientific data does not support mHealth apps developed specifically for children [30, 48]. However, the most sought-after features of mHealth apps developed for mental health are transparency and trust [49]. Therefore, healthcare professionals should evaluate the design quality and therapeutic and scientific accuracy of existing apps before integrating mHealth interventions into healthcare for patients with childhood anxiety disorders [44]. In addition, strategies to increase trustworthiness need to be developed for policymakers and healthcare professionals [48]. When navigating the mHealth app market, two common questions about quality and trust should be asked: “Which apps are effective?” and “How can I tell a good app from a bad one?” Healthcare professionals should follow mHealth app evaluation standards to answer these questions accurately [50].

## mHealth App Evaluation Standards

The use of mHealth apps in caring for people diagnosed with childhood stress disorders is increasing [45]. However, most apps are not adequately monitored for effectiveness, quality, safety, and privacy. The fact that user ratings do not reflect the clinical impact most are developed by investors, and potential data breaches increase the risks of unmonitored use [48, 50]. Therefore, healthcare professionals need to be able to evaluate mHealth apps for safety, effectiveness, and appropriateness before using them [47–49].

**Fig. 1:**
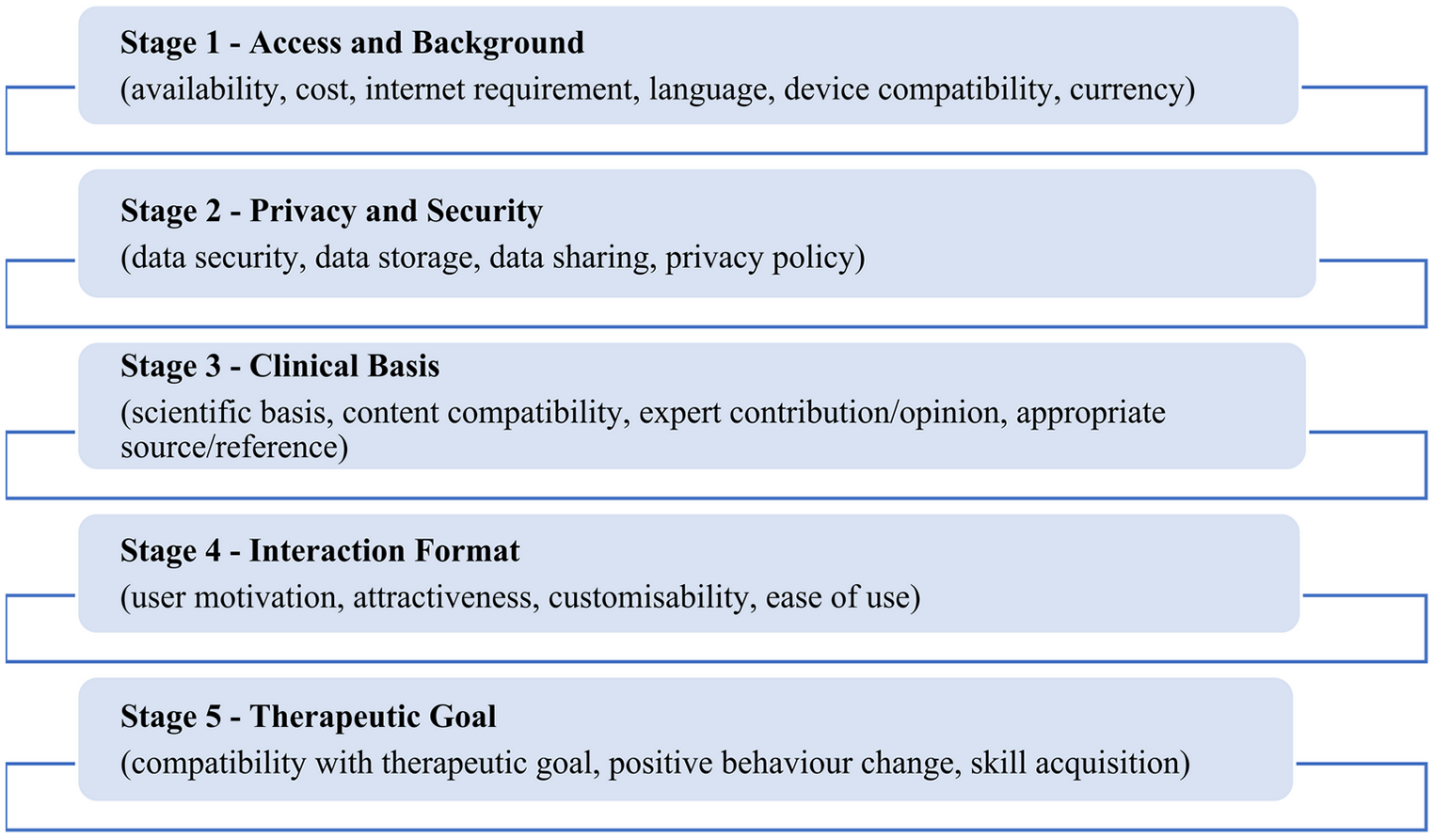
American Psychiatric Association App Evaluation Model Application Stages (Fig. 1 was created by the authors in alignment with existing literatüre [47–51])

Various guidelines, scales, and databases have been developed for this purpose. One of the most widely used is the ‘App Evaluation Model’ presented by the American Psychiatric Association in 2021. This model provides a five-step evaluation process to ensure a safe and personalized selection of mobile health apps [49, 51]. As shown in Fig. 1, the stages are designed to build progressively on one another and are marked by a patient-centered and flexible structure [47–51].

The scales used to evaluate mHealth applications are A-MARS (Adapted Mobile App Rating Scale) [56], ABACUS (App Behaviour Change Scale) [57], and THESIS [58]. A-MARS assesses user experience (functionality, aesthetics, etc.), ABACUS assesses behavior change potential, and THESIS assesses privacy, technical quality, and health content of mHealth applications developed for patients with chronic diseases. These scales are usually used in combination with other tools [47, 56–58].

One Mind PsyberGuide, one of the databases used to evaluate mHealth applications, focuses on mental health applications. It includes criteria such as scientific evidence, usability, and transparency. It also includes information on applications specifically targeted at young users. Recommendations are differentiated for adult and young users [49, 53, 59]. Another database, MIND (mHealth Index and Navigation Database), includes many applications based on the APA framework. Applications are rated under many headings, such as privacy, data use, user control, and clinical evidence. It also includes applications for children. It also allows the assessment of input/output characteristics of mHealth applications [52, 53, 60].

In summary, evaluation tools for mobile applications have been developed for children. However, most mHealth applications are aimed at general users. Therefore, parents, clinicians, and researchers need to evaluate applications with additional eyes, especially in terms of privacy, appropriateness of content, and scientific evidence [30]. General principles for the effective use of mHealth applications in children are listed below [30, 44].

- mHealth applications should be evaluated with appropriate tools before being recommended to children and selected from appropriate databases such as MIND [13, 44].
- mHealth applications should provide additional legal protection for children’s data. They should also be appropriate regarding language, culture, cost, and infrastructure [15, 30].
- mHealth applications should be appropriate for the age and developmental level of the child. Voice prompts and visual guides are necessary for children with limited literacy skills [15, 30].
- mHealth applications should support parental involvement and include coaching or follow-up functions [13, 34].
- mHealth applications should support specialized applications such as exposure therapy [15, 34].
- Applications should be regularly updated [44].
- Education should include evidence-based information and be fun, simple, and interactive [13, 30].

## Data Security and Privacy in mHealth Applications

The number of mobile applications developed in the mental health field is increasing daily, and it is becoming increasingly complex for mental health professionals and clients to choose reliable and ethical applications. Although attention is paid to the usability of mobile applications, the issue of privacy is rarely addressed [61]. In particular, it is important to provide clear information about how user data is collected and protected within the application [62].

A study of applications developed for depression found that less than half of the applications had a privacy policy and inadequate data security [63]. It is also emphasized that applications should inform users according to the principle of transparency. Furthermore, how notifications are handled in mobile applications is particularly important [64]. In particular, the appearance of mental health information on the screen may lead to an invasion of privacy by third parties. Therefore, it is important to inform the user about the content of application notifications and establish open communication with the client about privacy [64]. In this context, in addition to the technical aspects of privacy, ethical responsibilities and regulations regarding user rights should also be evaluated.

## Privacy and Data Management

The protection of client data is ethically important in psychological help services. The protection of data such as age, gender, and phone number of users in mobile applications against digital theft is considered to be a privacy issue [65]. Users have the right to know how their data is collected and for what purposes it is used. However, as many mobile applications do not have a privacy policy, data misuse is risky. Transparency by ethical and legal rules is therefore important [66]. It has also been observed that some applications access users’ email addresses and can use this information for advertising purposes [67]. The complex structure of digital platforms makes it even more challenging to ensure the security of user data. Therefore, applications incorporating technically strong data security measures supported by open protocols are needed [68]. In this sense, application developers and mental health professionals should also take responsibility for privacy and data management.

One issue that mental health professionals should be aware of is the security settings of mobile applications. They should examine an application’s privacy policy and ensure that its potential benefits outweigh the possible risks [67]. Therefore, the informed consent form should state the potential risks related to privacy that fall within the scope of the mobile application in the psychological counseling or psychotherapy process [65]. In addition to these technical and ethical issues of privacy and security, who and how applications can reach are also critical in providing equitable health services.

### Access and Equity in mHealth Applications

The equitable and inclusive use of mHealth applications in pediatric anxiety disorders remains limited due to several barriers. Several structural and social barriers exist to access and equity in mHealth applications. In particular, most commercial ones in application stores do not have scientific content [69], and privacy and security issues affect inclusivity. In addition, it is difficult to talk about equity in access to mHealth applications in regions with limited resources and socioeconomic difficulties [70]. Under these conditions, the cost-effectiveness and sustainability of applications remain uncertain.

The design, testing, and implementation of mHealth apps for anxiety disorders have been found to lack representation of immigrants, racial minorities, youth of color and cultural characteristics, Indigenous peoples, low socioeconomic status, and youth with diverse gender identities [13]. A recent study in early childhood found that the mHealth app was tested in a predominantly English-speaking and white region. Similar studies in the literature [67–69] also fail to provide sufficient data to increase inclusivity regarding ethnicity, socioeconomic status, or children with special needs. This suggests a significant lack of equitable representation in digital interventions.

Designing mHealth applications in only one language (usually English) can create barriers to use for people with different first languages. In addition, not taking into account cultural differences reduces user engagement and continuity with the application [74]. Furthermore, it is observed that mHealth designs are not designed to be usable and accessible for children with special needs (voice guidance, simple interface designs, visual-audio support, etc.), and these issues are generally ignored [75].

It is emphasized that diversity, equity, and inclusion (DEI) principles should be considered in developing mHealth applications in pediatric anxiety management [13]. These principles include representation of diverse groups of young people, co-production processes, and accessibility of applications at all stages. In addition, it is recommended that an intersectional approach be used to consider inclusivity when evaluating effectiveness [76]. Access and equity in mHealth is not only technical but also ethical and social responsibility, and developers, clinicians, researchers, and policymakers should work together in this direction. To address issues of access and representation, design approaches are needed to make mHealth applications more inclusive and user-centered.

## Recommendations for Inclusion

Recommendations to enhance inclusivity in mHealth applications for pediatric anxiety disorders are presented under three subheadings: Personalized and context-aware applications, ınclusive and participatory design, and trust: transparency, participation, and ınclusion.

### Personalised and Context-Aware Applications

The flexibility of mHealth applications, their adaptability to individual characteristics, and the personalization of treatment and duration are of great importance, especially for mental health conditions such as anxiety. This is supported by methodological models such as Multi-stage Optimisation Strategies (MOST) and Sequential, Multiple Assignment, Randomised Trials (SMART) [77, 78]. mHealth applications for mental health conditions such as childhood anxiety should be designed to take into account social characteristics (race, etc.) as well as differences in the child’s gender, family structure, interests, and social support [13]. Designing and implementing intersectional individual differences at the individual and community levels can ensure that mental health services are more inclusive and effective.

### Inclusive and Participatory Design

In mHealth applications developed for mental health problems such as anxiety, human- and community-centered designs facilitate understanding of user needs and early identification of potential barriers to implementation [79, 80]. Sensitivity to cultural differences in the design process requires consideration of structural barriers such as lack of trust, access to limited resources, and stigma, particularly in selecting participants [81, 82].

Inclusivity is strengthened by individual and community-based approaches to engage underrepresented groups in the process [83]. In the BlueIce application developed with this understanding, young people were actively involved in the co-design process and provided feedback on content, design, and visual elements. The application has been associated with reductions in anxiety, depression, and self-harming behaviors and has been highly adopted by users [84]. Similarly, the Momentum application was designed with input from young people, families, and experts to support the mental health of people aged 7–17. It provides a user-centered and culturally appropriate experience with an age-appropriate, customizable structure [85]. These examples show that participatory design is important in increasing user engagement in mHealth. Therefore, participatory approaches should be adopted as a standard design principle in mHealth applications.

### Trust: Transparency, Participation, and Inclusion

Although applications developed for mental health issues such as anxiety are accessible, if they do not adequately represent the community, they will not inspire trust and prevent the adoption of the application [86]. Therefore, the design of mHealth interventions should be based on trust. Involving participants in the process from the early stages of design will increase trust and ensure the adoption of the application [87].

It is important that applications are designed with transparency and ethical considerations in every aspect and that they can be integrated into communities, schools, or healthcare settings beyond individual use [88]. In this way, it contributes to the widespread acceptance of mHealth applications in the management of anxiety disorders that are common in society. Building trust through inclusive design, transparency, and ethical integration is essential in mHealth development for pediatric anxiety. To consolidate the dimensions discussed across the review, Table 1 summarizes key criteria for evaluating such applications regarding clinical relevance, accessibility, and safety.

**Table 1:** Key evaluation dimensions in mHealth applications for pediatric anxiety disorders

Evaluation Category	Description / Scope	Example / Source
Content Appropriateness	Age and developmental suitability, gamification	SmartCAT 2.0, Lumi Nova
Parental Involvement	Structured features enabling parent participation, psychoeducation, feedback, goal-setting, and homework support	Rational Parenting Coach (RPC), SPACE Program
Evidence-Based Approach	Use of CBT/exposure-based content, scientific structure grounded in psychotherapy models	MindClimb, Anxiety Coach
Data Security and Privacy	Privacy policies, transparency in data collection and processing, secure communication	CDC, APA Framework
Accessibility and Inclusivity	Accessibility regarding language, culture, socioeconomic status, and special needs	Momentum, BlueIce
Effectiveness and Efficacy	Evidence from RCTs, user satisfaction, long-term impact	Systematic Reviews, Meta-analyses
Quality Assessment Tools	Use of evaluation frameworks such as MIND, PsyberGuide, A-MARS, THESIS, and ABACUS	APA, OneMind, MIND Database

Table 1 was created by the authors in alignment with existing literature [12, 14, 29, 39, 45, 47, 56–58]

## Conclusions

mHealth applications developed for childhood anxiety disorders offer an important opportunity to overcome the limitations of traditional therapy methods. Although there is evidence in the literature to support the effectiveness of exposure-based CBT, integrating this approach into digital interventions is not strong enough. In addition, most of the existing applications lack a scientific basis and do not adequately meet the needs of children. There is a need for further standardization and evaluation of effectiveness, data security, and accessibility. In this context, mHealth applications with evidence-based content and design that are culturally and age-appropriate, user-centered, and developed through multi-stakeholder collaborations should be evaluated in future studies using high-evidence research methods. In this way, mHealth applications can also be an important technological product regarding social justice and health equity.

## Key References

- Dülsen P, Baumeister H. Internet- and mobile-based anxiety and depression interventions for children and adolescents: efficacy and negative effects—a systematic review and meta-analysis. Eur Child Adolesc Psychiatry. 2025;34(1):101–121. doi:10.1007/s00787-024-02270-7.
-
  - A recent meta-analysis examining both the effectiveness and potential adverse effects of mobile mental health interventions in children and adolescents.
- Lockwood J, Williams L, Martin JL, et al. Effectiveness, user engagement and experience, and safety of a mobile app (Lumi Nova) delivering exposure-based cognitive behavioral therapy strategies to manage anxiety in children via immersive gaming technology. JMIR Ment Health. 2022;9(1):e29008. doi:10.2196/29008.
-
  - Evaluates a child-focused, exposure-based CBT app using gamification, showing promising outcomes in anxiety reduction.
- Mescher T, Hacker RL, Martinez LA, et al. Mobile health apps: guidance for evaluation and implementation by healthcare workers. J Technol Behav Sci. 2024. doi:10.1007/s41347-024-00441-7.
-
  - Provides practical guidance for healthcare professionals on assessing and implementing mHealth apps safely and effectively.
- American Psychiatric Association. The App Evaluation Model [Internet]. Washington, DC: APA; [cited 2025 Apr 7]. Available from: https://www.psychiatry.org/psychiatrists/practice/mental-health-apps/the-app-evaluation-model.
-
  - This model offers a step-by-step framework for clinicians to evaluate mental health apps based on privacy, evidence, and engagement.
- MIND: mHealth Index and Navigation Database [Internet]. American Psychiatric Association; [cited 2025 Apr 7]. Available from: https://apps.digitalpsych.org/.
-
  - A comprehensive database that helps professionals assess mHealth apps using standardized quality metrics aligned with APA’s model.
- One Mind PsyberGuide [Internet]. Irvine (CA): One Mind; [cited 2025 Apr 7]. Available from: https://onemindpsyberguide.org/.
-
  - A non-profit project that rates mental health apps based on credibility, transparency, and user experience, tailored for both adults and youth.
- Giovanelli A, Karver TS, Roundfield KD, et al. The Appa Health app for youth mental health: Development and usability study. JMIR Form Res. 2023;7(1):e49998. 10.2196/49998.
-
  - Describes the development and usability testing of a youth-oriented mHealth application that incorporates therapeutic principles.
- Litke SG, Resnikoff A, Anil A, et al. Mobile technologies for supporting mental health in youths: scoping review of effectiveness, limitations, and inclusivity. JMIR Ment Health. 2023;10(1):e46949. doi:10.2196/46949.
-
  - A comprehensive review that explores both the promise and limitations of mobile mental health technologies for young populations.
- Magomedova A, Fatima G. Mental health and well-being in the modern era: a comprehensive review of challenges and interventions. Cureus. 2025;17(1):e77683. doi:10.7759/cureus.77683.
-
  - Highlights systemic and design challenges in digital mental health and explores opportunities for inclusive, ethical innovation.

## Data Availability

No datasets were generated or analysed during the current study.
